# Induction of p21^CIP1/WAF1 ^expression by human T-lymphotropic virus type 1 Tax requires transcriptional activation and mRNA stabilization

**DOI:** 10.1186/1742-4690-6-35

**Published:** 2009-04-08

**Authors:** Ling Zhang, Huijun Zhi, Meihong Liu, Yu-Liang Kuo, Chou-Zen Giam

**Affiliations:** 1 Department of Microbiology and Immunology, Uniformed Services University, 4301 Jones Bridge Road, Bethesda, MD 20814, USA

## Abstract

HTLV-1 Tax can induce senescence by up-regulating the levels of cyclin-dependent kinase inhibitors p21^CIP1/WAF1 ^and p27^KIP1^. Tax increases p27^KIP1 ^protein stability by activating the anaphase promoting complex/cyclosome (APC/C) precociously, causing degradation of Skp2 and inactivation of SCF^Skp2^, the E3 ligase that targets p27^KIP1^. The rate of p21^CIP1/WAF1 ^protein turnover, however, is unaffected by Tax. Rather, the mRNA of p21^CIP1/WAF1 ^is greatly up-regulated. Here we show that Tax increases p21 mRNA expression by transcriptional activation and mRNA stabilization. Transcriptional activation of p21^CIP1/WAF1 ^by Tax occurs in a p53-independent manner and requires two tumor growth factor-β-inducible Sp1 binding sites in the -84 to -60 region of the p21^CIP1/WAF1 ^promoter. Tax binds Sp1 directly, and the CBP/p300-binding activity of Tax is required for p21^CIP1/WAF1 ^trans-activation. Tax also increases the stability of p21^CIP1/WAF1 ^transcript. Several Tax mutants trans-activated the p21 promoter, but were attenuated in stabilizing p21^CIP1/WAF1 ^mRNA, and were less proficient in increasing p21^CIP1/WAF1 ^expression. The possible involvement of Tax-mediated APC/C activation in p21^CIP1/WAF1 ^mRNA stabilization is discussed.

## Background

Human T cell lymphotropic virus type 1 (HTLV-1) is the etiologic agent of adult T-cell leukemia/lymphoma and tropical spastic paraparesis/HTLV-associated myelopathy. HTLV-1 encodes a 40 kDa trans-activator, Tax, which plays a crucial role in viral replication and cell transformation [1-3]. Tax activates the expression of viral and cellular genes by interacting with a variety of host cell factors [4,5] including transcription factors CREB/ATF [6-9], transcriptional co-activators CBP/p300 [10-14], and the regulatory subunit of the I-κB kinase, IKKγ [15-20].

Previous studies have indicated that Tax causes many eukaryotic cells to develop mitotic abnormalities [21,22]. We have found recently that Tax can commit eukaryotic cells into a senescence-like state with dramatically up-regulated expression of p21^CIP1/WAF1 ^(p21) and p27^KIP1 ^(p27) [23]. More recently, we have found that both transduction of the *tax *gene and infection with HTLV-1 can cause HeLa cells and SupT1 cells to become senescent or cell cycle arrested with greatly elevated levels of p21 and p27 [24]. The sharp rise in p27 induced by Tax is due to the aberrant activation of a critical E3 ubiquitin ligase, the anaphase promoting complex/cyclosome (APC/C), which controls mitotic progression and exit [23,25-27]. The prematurely activated APC/C causes Skp2, the substrate-targeting subunit of another E3 ubiquitin ligase, SCF^Skp2^, to be degraded during S phase. The loss of Skp2 leads to the inactivation of SCF^Skp2 ^and the dramatic stabilization of p27 [23], a key substrate of SCF^Skp2 ^[28-30].

P21 was initially characterized as an important inhibitor of cyclin/Cdk2 complexes [31]. It becomes induced by the tumor suppressor p53 in response to DNA damage and arrests the cell cycle at the G_1_/S checkpoint [32-35]. Subsequent studies have revealed that p21 can bind the proliferating cell nuclear antigen (PCNA) and further inhibits DNA synthesis during S phase [36]. As a potent inhibitor of G1/S Cdks and DNA replication, p21 plays an important role in terminal differentiation and senescence [33-35]. It can confer protection from apoptosis [37]. Paradoxically, p21 also serves as a platform for the interaction between cyclin D and Cdk4/6, and promotes the assembly of active G1 Cdk complexes [33].

A wide variety of mechanisms including transcriptional regulation, mRNA degradation, ubiquitin-dependent or ubiquitin-independent proteolysis, and subcellular localization are known to regulate the level and activity of p21 [38]. At the transcriptional level, even though tumor suppressor p53 is a major trans-activator of p21, other transcription factors such as Sp1/Sp3, E2F, Smads, AP2, CAAT/enhancer-binding proteins, signal transducers and activators of transcription (STAT) and BRCA1 also regulate p21 expression [35]. It has been shown previously that Tax functionally inactivates p53 [39]. Since p53 plays a major role in the expression of p21, the dramatic up-regulation of p21 by Tax would appear to be affected by a p53-independent mechanism. Indeed, earlier studies have indicated that to be the case [40]. Here we show that the Tax-responsive element in the p21 promoter resides in two Sp1-binding sites (in the -84 to -60 region) previously shown to mediate the induction of p21 expression by tumor growth factor β (TGF-β) [41]. Trans-activation of these regulatory elements by Tax and by Sp1 are additive. Our data suggest that Tax directly tethers Sp1 and the ability of Tax to interact with CBP/p300 is important for p21 promoter trans-activation.

Transcriptional activation of the p21 promoter alone, however, cannot fully explain the dramatic increase in p21 brought about by Tax. We provide evidence to show that p21 mRNA stability is greatly increased in the presence of Tax. Analyses of newly isolated Tax mutants suggest that the ability of Tax to activate APC/C also correlates with p21 mRNA stabilization. These results reveal a hitherto unknown property of Tax in regulating p21 mRNA turnover.

## Results

### Identification of the Tax-responsive element in the p21 promoter

Previous studies have shown that HTLV-1 Tax can trans-activate the p21 promoter independently of the tumor suppressor, p53 [40]. The *cis *elements in the p21 promoter responsible for Tax trans-activation have not been well defined, however. To this end, we obtained a full-length p21 promoter-luciferase reporter plasmid (p21) and a derivative (p21Δ1) that contains a truncated p21 promoter, which begins at 298 nucleotides upstream (-298) and ends at 16 nucleotides downstream (+16) of the p21 mRNA start site. We also constructed two p21 promoter deletions with progressively shortened DNA sequences (100 and 60 bps) upstream of the p21 transcriptional start site. These deletions were inserted upstream of the firefly luciferase gene to produce p21Δ2, and p21Δ3 respectively. Each of these reporter plasmids was transfected into HeLa/18 × 21-EGFP cells together with a Tax expression plasmid, pBC12-Tax. The luciferase reporter activities were then measured 48 h after transfection.

**Figure 1:**
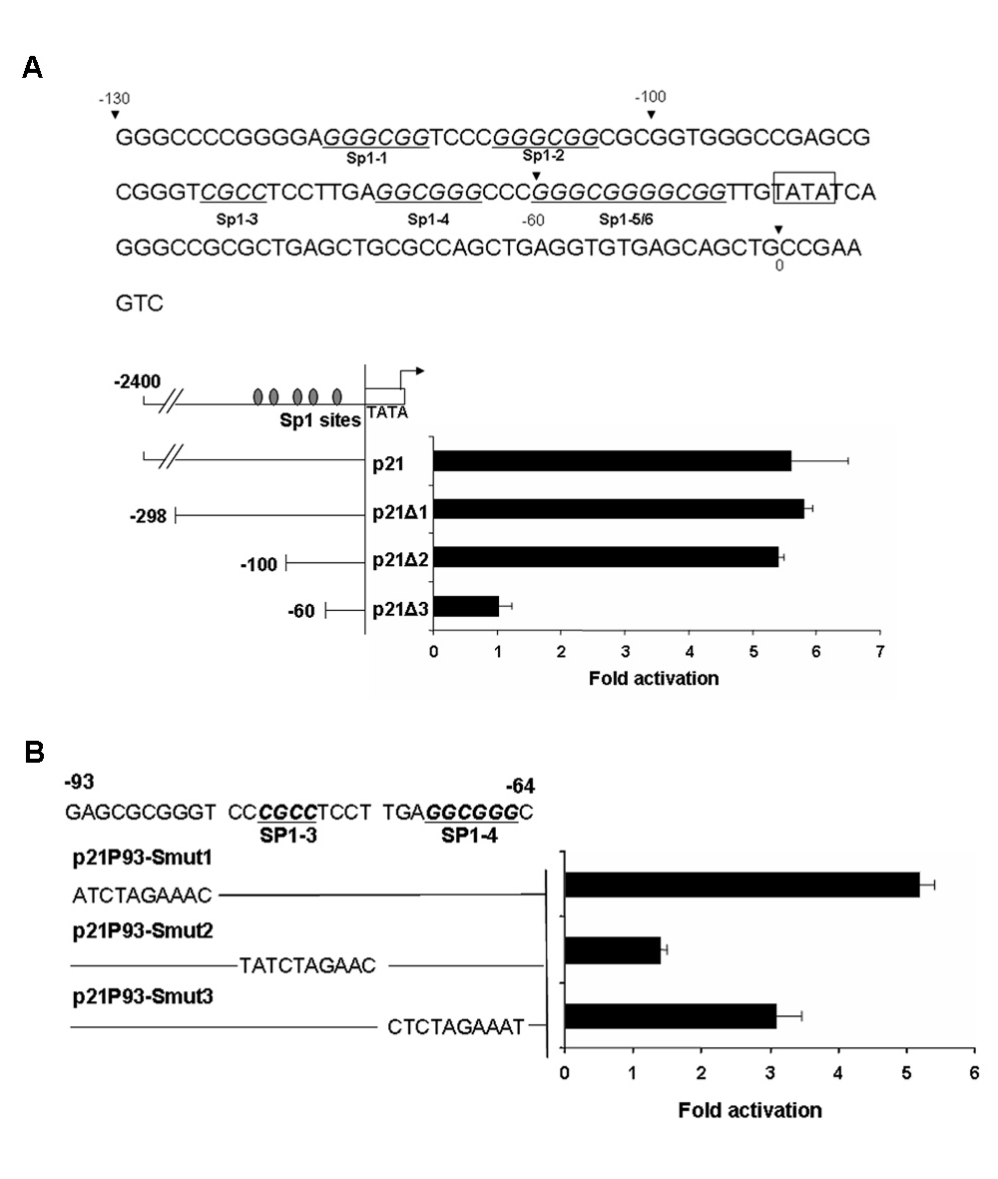
**Identification of the Tax-responsive elements in the p21 promoter**. *A*, The DNA sequence of the region covering 130 nucleotides upstream and 8 nucleotides downstream of the p21 mRNA start site (is shown (Top). The p21-Luc reporter plasmid contains a 2342 bp DNA fragment that spans 2326 bp upstream and 16 bp downstream of the p21 transcriptional start site fused to the firefly luciferase reporter gene. The p21 promoter truncation p21Δ1, p21Δ2, and p21Δ3 have their 5' termini ending at 298, 100, and 60 upstream of the p21 transcriptional start site (see METHODS). HeLa/18 × 21-EGFP cells were transfected in triplicate with the respective luciferase reporter constructs with or without a Tax expression plasmid, BC12-Tax. Cell lysates were prepared 48 h after transfection for luciferase assays as previously described [59]. All firefly luciferase reporter assays were normalized against HSV thymidine kinase (TK) promoter-driven renilla luciferase reporter activities. Standard deviations of the reporter activities and the degree of trans-activation by Tax (fold activation) are shown. *B*, TATA-box proximal Sp1-binding sites in the p21 promoter mediate Tax trans-activation. Luciferase reporter assays were as in *(A)*. The constructs p21P93-Smut-1, 2, and 3 contain base substitutions in the Sp1 binding sites localized in -93 to -84, -83 to -74, and -73 to -64 regions of the p21 promoter respectively.

As shown in Fig. 1A, Tax trans-activated the reporter containing the full-length p21 promoter approximately five-fold. Mutants p21Δ1 and p21Δ2, which contained 298 and 100 bp upstream of the p21 transcriptional start site respectively, were activated by Tax to similar extent as the full-length promoter. Since the p53-response elements are localized in the -2285 and -2255 region of p21 promoter, our results are consistent with previous reports that activation of p21 promoter by Tax is p53-independent [40]. Importantly, the deletion containing only the -60 sequence of the p21 promoter completely lost Tax responsiveness, indicating that the *cis *element responsible for the transcriptional induction by Tax resides between the -100 to -60 region. Interestingly, previous studies have shown that two binding sites for the Sp1 family of transcriptional factors reside within the sequence between -83 and -64, and this region mediates up-regulation of p21 transcription in response to tumor growth factor β (TGF-β) and arsenic compounds [41].

To further define the *cis *element responsible for Tax induction, we analyzed three mutants: p21P93-S-mut-1, mut-2 and mut-3, each containing ten consecutive base substitutions in the -93 to -84, -83 to -74, and -73 to -64 sequences, with the latter two mutations spanning the Sp1-binding sites Sp1–3 and Sp1–4 respectively. As indicated by reporter assays, base substitutions in the sequence between -93 and -84 had no effect on Tax induction. By contrast, p21P93-S-mut-2 completely lost Tax responsiveness, while p21P93-S-mut-3 was partially impaired (Fig. 1B). These results indicate that Sp1–3, and to a lesser extent, Sp1–4, is critical for Tax-mediated activation of the p21 promoter. Interestingly, the Tax-responsive element coincided exactly with the TGF-β responsive element previously identified [41].

### Trans-activation of the p21 promoter by Tax and Sp1 are additive

**Figure 2:**
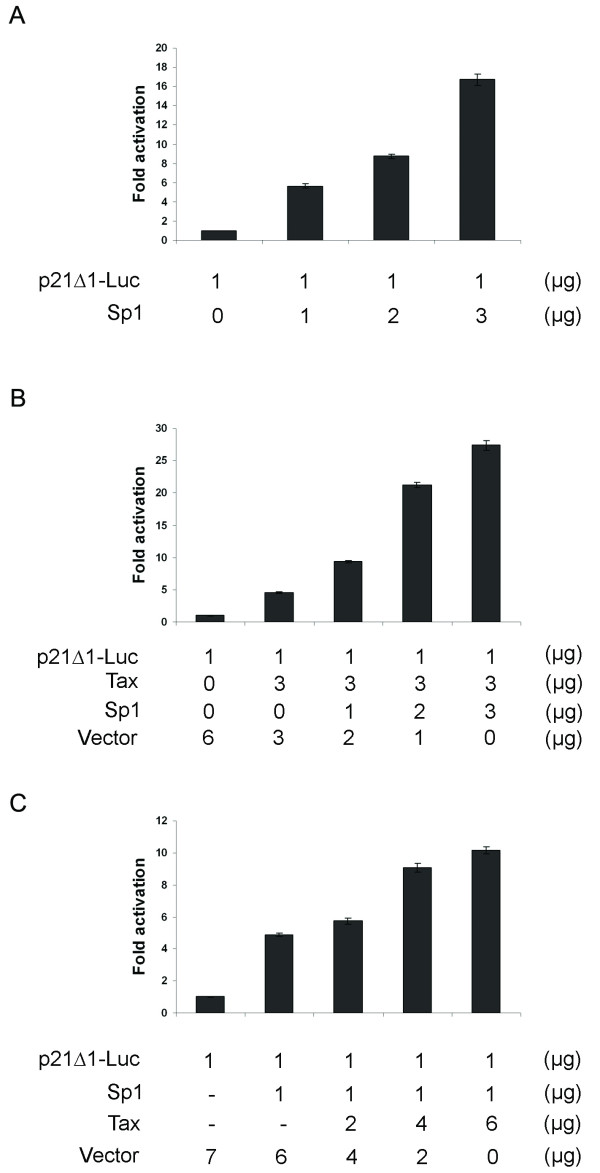
**Trans-activation of the p21 promoter by Tax and Sp1 is additive**. *A*, HeLa/18 × 21-EGFP cells were transfected with 1 μg of p21Δ1-Luc either alone or in combination with increasing amounts (1, 2, or 3 μg) of an Sp1 expression plasmid, Flag-Sp1. Luciferase activity was measured and quantified as in Fig. 1A. B, HeLa cells were transfected with 1 μg p21Δ1-Luc and 3 μg pBC12-Tax without or with increasing amounts of the Flag-Sp1 plasmid. The total amount of transfected DNA in each well was kept at 7 μg. *C*, HeLa cells were transfected with 1 μg each of the p21Δ1-Luc and the Flag-Sp1 plasmid without or with 2, 4, 6 μg of pBC12-Tax plasmid. The total amount of transfected DNA in each well was kept constant at 8 μg by the addition of an empty vector plasmid. The fold trans-activation of each effector is plotted as above.

Because Sp1 had been shown earlier to mediate TGF-β induction of p21 expression [1,42], we examined if Sp1 is also involved in Tax-mediated trans-activation of the p21 promoter. In agreement with published literature, transfection of an expression construct for Sp1, Flag-Sp1, activated the p21 promoter in a dose-dependent manner (Fig. 2A). Co-transfection of the p21Δ1-Luc reporter with a fixed amount of Tax-expression construct, pBC12-Tax, and increasing amounts of Flag-Sp1 indicated that Tax functions additively with Sp1 to augment luciferase gene expression driven by the p21 promoter (Fig. 2B). Similar results were obtained when a constant amount of Flag-Sp1 DNA and increasing amounts of pBC12-Tax were used (Fig. 2C). These results suggest that Tax and TGF-β possibly act through distinct mechanisms to activate p21 expression.

### Tax does not alter Sp1 expression but interacts with Sp1 directly

**Figure 3:**
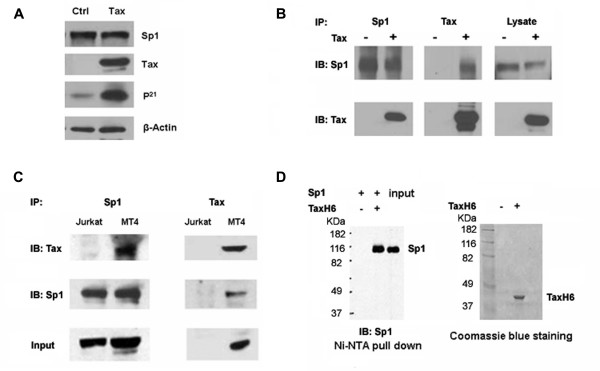
**Direct interaction between Tax and Sp1**. *A*, Tax does not alter Sp1 level. HeLa/18 × 21-EGFP cells were transfected with a control plasmid, pcDNA3 (Ctrl), or pBC12-Tax (Tax), using the calcium phosphate method. Immunoblotting was used to detect Sp1, Tax, p21, and β-Actin in the whole cell lysates at 48 h post-transfection. *B*, Tax associates with Sp1 in transfected HEK293 cells. HEK293 cells were transfected with pcDNA3 (-) or pBC12-Tax (+) as in (*A*). Cell lysates were immunoprecipitated with Sp1 or Tax antibody and immunoblotted with the same. *C*, Tax associates with Sp1 in HTLV transformed MT4 T cells. Jurkat and MT4 cell lysates were immunoprecipitated with Sp1 or Tax antibody and immunoblotted with the same as indicated. *D*, Tax binds Sp1 *in vitro*. Approximately 200 ng of Sp1 (Promega), and 30 μl of Ni-NTA agarose beads were mixed with (+) or without (-) purified hexa-histidine-tagged Tax (TaxH6) (~2 μg) as described in METHODS. The beads were collected by centrifugation and washed with the binding buffer. The bound proteins were eluted in SDS sample buffer and analyzed by immunoblotting (Sp1) and Coomassie blue staining (Tax).

Since the Sp1-binding sites in the p21 promoter are directly responsible for Tax-mediated trans-activation and Tax is not known to bind the Sp1-binding sites, we tested if Tax might increase the level of Sp1 expression. To this end, a HeLa cell line containing a Tax-responsive reporter cassette, 18 × 21-EGFP, was transfected with the pBC12-Tax plamid. Fluorescence microscopy indicated that greater than 80% of HeLa cells had been transfected and became EGFP-positive (not shown). The Sp1 level in the transfected cells, however, was unchanged when compared to that in cells transfected with a control plasmid (Fig. 3A), indicating that Tax had no effect on Sp1 level. We next tested if Tax directly interacted with Sp1 *in vivo *and *in vitro*. Sp1 and Tax were found to co-immunoprecipitate from whole cell lystates of pBC12-Tax-transfected HEK293 cells (Fig. 3B), and HTLV-1-transformed human T cell line, MT4 (Fig. 3C), but not from lysates of control HEK293 or HTLV-unrelated Jurkat T cells. Finally, purified Sp1 protein could bind hexa-histidine-tagged Tax immobilized on a metal-chelating column (Fig. 3D).

### Activation of the p21 promoter by Tax requires CBP/p300 binding, but promoter trans-activation is not sufficient to fully up-regulate p21 expression

Taking advantage of the ability of Tax to cause cell cycle arrest in *S. cerevisiae*, we have isolated and characterized *tax *mutants whose expression in *S. cerevisiae *cells did not lead to cell cycle arrest. Many of these mutants, including L235F and A108T, remain capable of trans-activating both the HTLV-LTR-Luc and the NF-κB-Luc reporters, but are attenuated in induction of p21 and p27, and cell cycle arrest/senescence in HeLa cells. These mutants were previously found to be deficient in activating the anaphase promoting complex/cyclosome (APC/C) [27].

**Figure 4:**
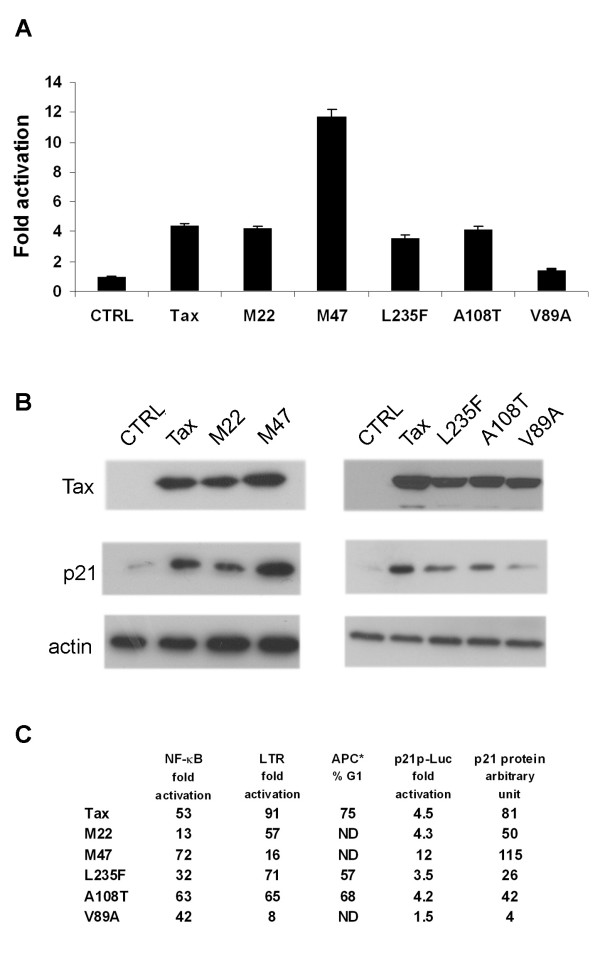
**Trans-activation of the p21 promoter is necessary but not sufficient to increase p21 expression**. *A*, HeLa/18 × 21-EGFP cells were transfected with p21Δ1-Luc without or with 3 μg of each of the expression plasmids for wild-type and mutant Tax (M22, M47, L235F, A108T, V89A) as in Fig. 1. The reporter assays and analyses are as in Fig. (1). *B*, Twenty micrograms of total cell proteins from each transfection were resolved in a 12%SDS-polyacrylamide gel and immunoblotted using the 4C5 mouse hybridoma antibody against Tax, the rabbit anti-p21 antibody, and the rabbit anti-β-actin antibody respectively. *C*, A Summary the biological properties of various Tax alleles. NF-κB, LTR, and p21p-Luc fold transactivation shows the fold activation of the E-selectin-Luc, HTLV-1 LTR-Luc, and the p21 proximal promoter-Luc reporters by wild-type *tax *(Tax) or the respective mutant allele (M22, M47, L235F, A108T, and V89A) is indicated. APC % G1: The fractions of HeLa cells in G1 after transduction by wild-type *tax *(Tax) or mutant *tax *allele (L235F and A108T). ND denotes "not determined". p21 protein arbitrary unit indicates the ratio of the level of p21 protein normalized again that of Tax after densitometer scanning. The p21 level in M47 is likely underestimated because of the non-linearity of film over-exposure to the immunoblot. The mRNA of p21 is significantly stabilized by M47 as shown in Fig. 6.

To further elucidate the mechanism by which Tax up-regulates p21 expression, we analyzed APC/C-deficient mutants L235F and A108T along with three well-characterized *tax *mutants: M22, M47, and V89A that had been shown to be impaired, respectively, in NF-κB activation, LTR trans-activation, and CBP/p300-binding and LTR trans-activation [12]. As shown in Fig. 4A, all mutants except the CBP/p300-binding-deficient V89A were able to up-regulate the p21 promoter to the same extent as the wild-type *tax*, suggesting that CBP/p300 recruitment is critical for the transcriptional activation of the p21 promoter by Tax. Interestingly, the M47 mutant, which remains capable of CBP/p300 binding but is defective in LTR activation, stimulated p21 promoter to a much greater extent than the wild-type Tax. These results support the notion that by directly interacting with Sp1, Tax may facilitate the recruitment of CBP/p300 to the p21 promoter to activate transcription.

Activation of the p21 promoter alone, however, is not ultimately sufficient to account for the Tax-induced increase in p21 level. Tax mutants, M22, L235F, and A108T trans-activated the p21 promoter to levels comparable to that of the wild-type Tax, yet were attenuated in elevating the p21 protein level (Fig. 4A and 4B). This, together with the fact that Tax causes a 40 fold increase in p21 mRNA level [23], but only activated p21 promoter 5 fold, suggest that post-transcriptional regulation plays an important role in Tax-mediated increase of p21 (Fig. 4B). The reporter activities, the proficiency in causing G1 arrest, and the extent p21 of induction of the various Tax alleles are summarized in Fig. 4C[12,27,43].

### Tax stabilizes p27 but does not affect p21 protein turn-over

**Figure 5:**
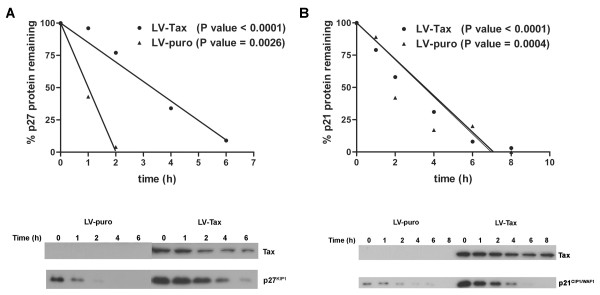
**Tax stabilizes p27 but not p21**. *A*, Tax increases the half-life of p27 protein. HeLa/18 × 21-EGFP cells were transduced with a lentiviral vector for Tax, LV-Tax, or control lentivirus LV-puro for 48 h. Cells were then treated with cycloheximide (100 μg/ml) for 0, 1, 2, 4, and 8 hours to inhibit *de novo *protein synthesis and harvested for p27 immunoblots. The level of p27 at each time point was quantified by the Scion Image software. The level of p27 at time 0 was set as 100%. The percent p27 protein remaining at each time point was calculated accordingly, and then plotted. Linear regression analysis of each data set was carried out using the Prism software package. The *P *value of each line is as indicated. *B*, Tax has no effect on p21 protein turn-over. The experiment was carried out and analyzed similarly as in *(A) *except that the rate of p21 turn-over was examined.

We have shown recently that the premature activation of the mitotic E3 ubiquitin ligase, the anaphase promoting complex, by Tax leads to the polyubiqutination and degradation of Skp2, and consequently, inactivation of the G1/S E3 ubiquitin ligase, SCF^Skp2^. As a result, p27, a key substrates of SCF^Skp2^, becomes greatly stabilized when Tax is expressed, causing HeLa cells to enter into a state of irreversible G1 arrest termed Tax-induced rapid senescence (Tax-IRS) [23]. Based on a previous report that suggested that p21 might be a substrate of SCF^Skp2 ^[44], we initially attributed the concurrent and even more dramatic surge in p21 induced by Tax to the inactivation of SCF^Skp2^. To determine the rate of p21 and p27 protein turnover, HeLa/18 × 21-EGFP cells transduced with LV-Tax or a control lentivirus vector, LV-puro, were treated with cycloheximide to inhibit *de novo *protein synthesis. The abundance of p21 and p27 in transduced cells was then measured after the cessation of protein translation. As indicated and reported previously [23], the half-life of p27 in cells transduced by LV-Tax is considerably longer than that in cells transduced with LV-puro (Fig. 5A). A different result was obtained when the half-life of p21 was measured. As shown in Fig. 5B, the stability of p21 is not significantly altered by Tax (Fig. 5B). These results confirm the notion that p27 is a substrate of SCF^Skp2 ^and becomes stabilized as a consequence of premature activation of APC/C and inactivation of SCF^Skp2 ^by Tax. By contrast, even though the Tax-induced increase in p21 and p27 correlated with APC/C activation, the up-regulation of p21 by Tax is achieved by a mechanism independent of SCF^Skp2^- and ubiquitin-mediated protein turnover.

### Tax stabilizes p21 mRNA

**Figure 6:**
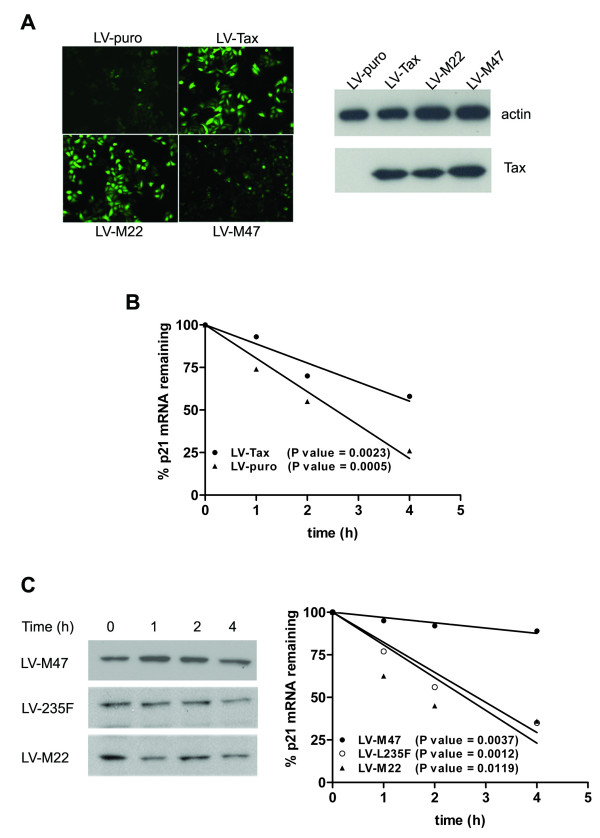
**Tax increases the half-life of p21 mRNA**. *A*, HeLa/18 × 21-EGFP cells were transduced with LV-puro, LV-Tax, LV-M22, and LV-M47 as described in METHODS. (Left panels) EGFP expression of cells transduced with the respective vectors. (Right panels) Levels of expression of various Tax alleles in transduced cells as determined by immunoblotting with the 4C5 Tax hybridoma antibody (Tax) and a control antibody against β-actin (actin). *B*, Tax stabilizes p21 mRNA. HeLa/18 × 21-EGFP cells were transduced with LV-Tax, or LV-puro for 48 h as in (*A*), and then incubated with actinomycin (2 μg/ml) for the indicated durations. The level of p21 mRNA in each sample was measured by real-time RT-PCR, quantified using the amount of the 18S rRNA as an internal reference, and finally, normalized to the p21 transcript level at time 0 and plotted and analyzed as in Fig. 5. *C*, APC/C-deficiency correlates with attenuation in p21 mRNA stabilization. (Left panel) HeLa/18 × 21-EGFP cells were transduced with LV-M47, LV-M22, and LV-L235F as in *(A)*. The rate of p21 mRNA turn-over in the transduced cells was determined as in (*B*). Data analysis and graphing were as described in Fig. 5 *A *(Right panels). Expression of the respective Tax alleles is as shown in the immunoblot.

The modest p21 promoter activation by Tax and the lack of increased p21 protein stabilization prompted us to examine the rate of p21 mRNA turnover by real-time PCR. To this end, the HeLa/18 × 21-EGFP cells were cultured in a six-well plate and transduced with LV-Tax for 48 h. Expression of the EGFP gene in HeLa/18 × 21-EGFP is driven by 18 copies of the Tax-responsive 21-bp-repeat. Therefore, the efficiency of *tax *gene transfer into HeLa/18 × 21-EGFP could be assessed by fluorescence microscopy. Care was taken to ensure that greater than 90% and comparable numbers of reporter cells were transduced with the respective lentivirus vectors (Fig. 6A, EGFP-positive cells, left panels). For the Tax mutant M47, which is impaired in activating the 18 × 21-EGFP cassette, in addition to the weaker EGFP signal detected, immunoblot of the transduced cells was also carried out to confirm that the level of M47 expression was equivalent to that of other Tax alleles (Fig. 6A right panels). The lentivirus vector-transduced cells were then treated with actinomycin D (2 μg/ml) to inhibit *de novo *mRNA synthesis. Total cellular RNA was prepared at 0, 1, 2, 4 h after actinomycin D treatment. Reverse transcription followed by quantitative real-time PCR was then used to measure the abundance of p21 mRNA at each time point. The relative level of p21 mRNA was then normalized against that of the 18S ribosomal RNA, further compared with the normalized p21 mRNA level at time 0, and plotted. As indicated in Fig. 6B, the half-life of p21 mRNA increased from 2.5 h to 4.5 h in the presence of Tax. Based on the length of the HeLa cell cycle of approximately 16–18 h, the amplification of p21 mRNA through this mechanism is likely to be in range of more than 10 fold within one cell cycle. This, coupled with the 5-fold induction of p21 promoter, could explain the increase in p21 mRNA transcript induced by Tax[23].

### Characterization of Tax mutants with altered abilities in stabilizing p21 mRNA

To confirm the importance of mRNA stabilization in Tax-mediated up-regulation of p21, the effect of Tax mutants M22, M47, and L235F on p21 mRNA half-life was further examined. HeLa/18 × 21-EGFP cells were transduced with the respective lentivirus vectors for 48 hours as in Fig. 6A. Immunoblot of L235F, M22, and M47 suggests that their levels of expression were comparable, with the level of M47 moderately higher (Fig. 6C left panels). As mentioned above, despite their wild-type-like competency in trans-activating p21 promoter, L235F and M22 were attenuated in up-regulating p21 (Fig. 4). Indeed, a direct measurement of the rate of p21 mRNA decay indicated that both L235F and M22 failed to stabilize p21 mRNA (Fig. 6C), with the p21 mRNA half-life of the respective transduced cells similar to that of the LV-puro-transduced control in Fig. 6B. Remarkably, the M47 mutant dramatically delayed the turnover of p21 mRNA (T_1/2 _≈ 9 h) and up-regulated p21 mRNA and protein expression to a much greater extent than the wild-type Tax (Fig. 6C right panel). Together, these results indicate that both promoter activation and mRNA stabilization are crucial for the sharp increase in p21 expression activated by Tax. Since L235F is attenuated in APC/C activation [27], this deficiency is likely responsible for the failure of L235F in increasing the stability of p21 mRNA transcript.

## Discussion

In this study, we have shown that two independent mechanisms: trans-activation and mRNA stabilization are responsible for the dramatic up-regulation of p21 by Tax. Our data are in agreement with other studies showing that Tax up-regulates p21 expression [40,45-49]. We have mapped the *cis*-elements in the p21 promoter that mediate Tax trans-activation to two Sp1-binding sites (-83 to -64) near the p21 mRNA start site. This region has been shown previously to be critical for TGF-β-mediated trans-activation of p21. Tax does not alter the intracellular level of Sp1. Rather it binds Sp1 directly. Analyses of Tax mutants further suggest that the interaction between Tax and CBP/p300 is important for p21 promoter trans-activation. Our results are consistent with a model in which Tax may serve as a tether between Sp1 and CBP/p300, and possibly help recruit the latter to the p21 promoter to activate transcription. These results are in agreement with a previous report that Tax can interact with Sp1 to activate c-sis/platelet-derived growth factor-B promoter [50]. Importantly, the 5-fold trans-activation of the p21 promoter cannot account fully for the dramatic 40-fold up-regulation of p21 by Tax [23]. Our data further indicate that Tax also induces post-transcriptional stabilization of p21 mRNA.

The level of p21, like that of p27, is cell cycle-regulated. It increases transiently after cellular exit from mitosis and entry into G1, and declines during G1/S transition. The increase in p21 and p27 after mitotic exit serves to maintain cells in G1 until such time when a new round of DNA replication is warranted. P21 also functions as a scaffold/chaperone for Cdk4-Cdk6/cyclin D assembly, thus helps set the stage for a new round of cell cycle. Since APC/C is intimately involved in regulating mitotic progression and mitotic exit, it is not surprising that the transient increase of p21 and p27 during early G1 is linked to the APC/C activity.

P27 is a substrate of the E3 ubiquitin ligase, SCF^Skp2 ^[28-30], which targets p27 for degradation. Recent evidence has indicated that Skp2 and another component of the SCF complex, Cks1, are both substrates of APC/C. During mitotic exit, APC^Cdh1 ^targets the degradation of Skp2 and Cks1, thereby rendering SCF^Skp2 ^inactive [51,52]. This in turn leads to the transient stabilization of p27 during G1. We have found that APC/C becomes activated by Tax in an unscheduled manner during S phase [23], thus causing premature degradation of a number of mitotic and cell cycle regulators including cyclin B1 and Skp2. The loss of Skp2 (and possibly Cks1) in turn causes Tax-expressing cells to accumulate p27 throughout S, G2, and M, leading to rapid senescence. Indeed, the half-life of p27 protein becomes greatly increased by Tax (Fig. 5A and ref. [23]).

Based on earlier reports that p21 may be a substrate of SCF^Skp2 ^[44], we originally thought that the dramatic increase in p21 induced by Tax is also mediated by the same mechanism as that for p27, i.e. through the inactivation of SCF^Skp2^. Contrary to earlier reports, our present data suggest that p21 is not a substrate of SCF^Skp2^, and the protein stability of p21, unlike that of p27, is not affected by Tax (Fig. 5B). Rather, in addition to the aforementioned p21 promoter trans-activation, Tax also causes p21 mRNA half-life to increase significantly. Our data further suggest that APC/C activation by Tax is responsible for the dramatic stabilization of p21 mRNA.

We have previously isolated several Tax mutants whose expression did not cause *S. cerevisiae *to undergo cell cycle arrest. Most of these mutants, including L235F and A108T, are attenuated in up-regulating p21 and p27 [27]. Importantly, they were found to be impaired in activating the APC/C [27]. As shown in Fig. 4, the inability of APC/C-deficient L235F and A108T mutants to fully up-regulate p21 is not due to a deficit in p21 promoter trans-activation. An in-depth analysis of L235F indicates that it is attenuated in stabilizing p21 mRNA (Fig. 6). Based on these results, we suggest that the stabilization of p21 mRNA during normal cell cycle progression or by Tax may be causally linked to the activity of the APC/C E3 ligase. Alternatively, since IKK-NF-κB activation and APC/C activation by Tax appear correlated [27], the stabilization of p21 may be a result of IKK or NF-κB activation. How APC/C activity or IKK/NF-κB activation leads to p21 mRNA stabilization mechanistically remains to be determined.

It has been reported previously that the 3' untranslated region (3'-UTR) of the p21 transcript contains AU-rich elements (AREs) that destabilizes p21 mRNA [53]. RNA-binding proteins of the HuR family including HuR and its homologues HuB, HuC, and HuD, on the other hand, bind AREs and prevent ARE-containing mRNAs such as that of p21 from degradation [54,55]. HuR proteins reside in the nucleus and their interaction with mRNA is facilitated by increased cytoplasmic localization of HuR or through post-translational modifications of HuR that increases its RNA binding affinity [56-58]. Whether Tax-APC/C-mediated and HuR-related mechanisms of p21 mRNA stabilization are connected is currently under investigation.

## Methods

### Cell culture

The HeLa/18 × 21-EGFP cell line was generated previously by transducing the parental HeLa cell line with a reporter cassette containing the EGFP gene under the control of 18 copies of the Tax-responsive 21-bp repeat element [59]. Cells were maintained in high glucose Dulbecco's modified Eagle's medium (DMEM) (Hyclone, Inc) supplemented with 10% fetal calf serum and 100 U/ml penicillin-streptomycin, and 2 mM L-glutamine. The HTLV-1-transformed T cell line MT4 was maintained in RPMI-1640 (Hyclone, Inc) containing the same supplements.

### Construction of the p21 Promoter Deletion Mutants

The full-length human p21 promoter construct, WWP-luc, was kindly provided by Dr. Wafik S EL-Deiry. It contains a DNA fragment encompassing 2.4 kb of the sequence upstream of the p21 mRNA start site. The p21Δ1 (REP-p21) construct was a generous gift of Dr. KJ. Zhao's at the NIH. It contains a truncated p21 promoter that begins at 298 nucleotides upstream (-298) and ends at 16 nucleotides downstream (+16) of the p21 mRNA start site. To construct p21Δ2, primers were designed to amplify the sequence between -100 to +16. The primer sequences were: 5'-AGTGCTAGCCGCGGTGGGCCGAGCGCG-3', and 5'-CCGAAGCTTAAGGAAC TGACTTCGGCA-3'. Nhe I and Hind III sites were incorporated at the ends of the PCR product, and the restriction endonucleases-digested DNA fragment was inserted into the REP-Luc construct. The p21Δ3 (-60 to +16) construct was made similarly except the PCR product was digested with Sma I and Hind III, and inserted into the Pvu II and Hind III sites of REP-Luc. Mutants of p21p93S mut1, 2, and 3 were generously provided by Dr. XF Wang.

### Transfection and luciferase Assay

The production of lentivirus vectors (LV-Tax and LV-puro) was as described previously [59]. For luciferase reporter assays, HeLa/18 × 21-EGFP cells were transfected with each of the reporter constructs with or without Tax using the calcium phosphate method. Cells were plated at a density of 1.0 × 10^5 ^cells/well in six-well plates one day before transfection. For each well, 5 μg of a plasmid encoding wild-type or mutant Tax and 0.5 μg of each of the p21 promoter constructs were used. To control for the variability in transfection, 0.5 μg of pRL-TK containing an HSV thymidine kinase (TK) promoter-driven *Renilla *luciferase reporter cassette (Promega) was included as an internal control. Cells were harvested 48 h after DNA transfection, and the firefly luciferase activity was assayed and normalized against *Renilla *luciferase activity. Luciferase assays were performed as previously reported [59].

### Tax-Sp1 co-immunoprecipitation

HEK 293 T cells grown to 40–50% confluence in a 100-mm tissue culture dish was transfected with 10 μg of a control plasmid, pcDNA3, or the Tax expression plasmid, pBC12-Tax, using the calcium phosphate method. Forty-eight hours post-transfection, cells were harvested by trypsinization and centrifugation at 14,000 × *g *for 5 min at 4°C. The cell pellet was washed once with 5 ml of cold PBS and resuspended for 15 minutes in 1 ml of lysis buffer (20 mM Tris-CI pH7.5, 100 mM NaCI, 1 mM EDTA, 1.5 mM MgCl_2_, 0.5% Triton-X100, 10 mM NaF, and 1 mM sodium orthovanadate) supplemented with a protease inhibitor cocktail (Roche Diagnostics). The cell lysates were cleared by spinning at 14,000 × *g *for 5 min at 4°C. The supernatant was pre-absorbed with 50 μl of a 50% slurry solution of immobilized rProtein G-Agarose (Invitrogen) for 1 h at 4°C. Thereupon, 400 μl of the supernatant was mixed with 2 μg of either a monoclonal anti-Tax (4C5) or a rabbit polyclonal anti-Sp1 (Santa Cruz Biotechnology, Inc) antibody. The samples were incubated for 1 h at 4°C with constant rotation, and 30 μl of a 50% slurry solution of rProtein G-Agarose was then added to each sample. The reaction mixtures were incubated for 2–16 h at 4°C with rotation. The beads were then gently washed 4–5 times with 500 μl of the lysis buffer. The bound proteins were eluted in SDS sample loading buffer, subjected to electrophoresis in a 10% denaturing polyacrylamide gel, transferred to nitrocellulose membrane, and immunoblotted for Tax and Sp1. Sp1-Tax co-immunoprecipitation from HTLV-1 transformed MT4 T cells, was carried out similarly as above except that 3 × 10^6 ^cells were used for each sample, and an HTLV-1-unrelated T cell line, Jurkat, was used as a control.

### Nickel-nitrilotriacetic acid (Ni-NTA)-agarose affinity pull-down

Approximately 200 ng of purified Sp1 (Promega) was mixed with 30 μl of Ni-NTA agarose beads in absence or presence of purified hexa-histidine-tagged Tax (TaxH6) (~2 μg) in 500 μl of binding buffer containing 20 mM Tris-Cl, pH 7.5, 100 mM NaCl, 1 mM EDTA, 0.5% Triton-X100, 100 mM imidazole, and 0.5 mg/ml BSA for 2 h at 4°C. The beads were collected by centrifugation and washed 3–4 times with 500 μl of the binding buffer without BSA. The bound proteins were eluted in SDS sample buffer and analyzed by immunoblotting (Sp1) or Coomassie blue staining (Tax).

### Protein and mRNA Half-life Determination

Around 1.5 × 10^5 ^HeLa/18 × 21-EGFP cells were seeded into each well of a six-well plate the day before treatment. LV-Tax and LV-puro were added at a titer of 5 × 10^5 ^infectious units/well for 48 h. For protein stability measurement, the transduced cells were treated with 100 μg/ml of cycloheximide for different times (0, 1, 2, 4, 6, and 8 h). The cells were then harvested, dissolved in SDS-sample buffer, and resolved with SDS-PAGE for immunoblottig with p21 and p27 antibodies. The intensities of the bands were determined by the Scion Image software and plotted using the Prism program.

For p21 mRNA half-life determination, the transduced cells were treated with actinomycin D (2 μg/ml) for different times (0, 1, 2, and 4 h) and harvested for RNA preparation. Total RNA was isolated using the TRIZOL Reagent kit (*Invitrogen*). To minimize genomic DNA contamination, about 20 μg of total cellular RNA was treated with 10 units of RNase-free DNase I at 37°C for 1 h. RNA was precipitated by adding one tenth volume of 3 M sodium acetate (pH 5.2), followed by the addition of 2 volumes of 100% ethanol, and kept frozen at -80°C for 1 h. The precipitated RNA was collected by centrifugation at 16,000 g for 20 min. Reverse transcription was performed using 1 μg total RNA in 10 μl of a reaction buffer from the Superscript First-strand Synthesis system (*Invitrogen*). For real-time PCR, the MicroAmp optical 96-well plates (Applied Biosystems) were used. One microliter cDNA template was added to each well, followed by the addition of 24 μl of a master mix containing 12.5 μl SYBER green (Applied Biosystems), 6.5 μl water, 2.5 μl (0.5 μM) each of the forward and reverse primer set. The 18S ribosomal RNA was used as an internal control to standardize the RT-PCR. Three replications were performed to ensure reproducibility of the measurements. The primers designed by the Primer Express software and are listed as follows: 18S rRNA forward primer: 5'-CGGCTACCACATCCAAGGAA-3' and 18S rRNA reverse primer: 5'-GCTGGAATTACCGCGGCT-3'; and p21 forward primer: 5'-CCATGTGGACCTGTCACTGT-3' and p21 reverse primer: 5'-TGGTAGAAATCTGTCATGCTGGTC-3'.

## Competing interests

The authors declare that they have no competing interests.

## Authors' contributions

LZ and C-ZG were responsible for the design of the study and the draft of the manuscript. LZ performed most of experiments. H-JZ performed Tax-Sp1 co-immunoprecipitation. M-HL and Y-LK provided reagents and cell lines, and assisted in interpretation of results. All authors read and approved the final manuscript.
